# Supplementary material for: Kinetochore-independent chromosome segregation driven by lateral microtubule bundles
Source: eLife. 2015 May 30;4:e06462. doi: 10.7554/eLife.06462 (PMC4481507; doi:10.7554/eLife.06462)
Supplement: Supplementary file 1. — Complete list of strains used in this study. DOI: http://dx.doi.org/10.7554/eLife.06462.020 [file elife06462s001.docx]

**SUPPLEMENTARY FILE 1:**

**Complete list of strains used in this study**

AV335 *emb-27(g48)II*; *unc-119(ed3) ruIs32[unc-119(+) pie-1^promoter^::GFP::H2B]III; ruIs57[unc-119(+) pie-1^promoter^::GFP::tubulin]*

(Wignall and Villeneuve, 2009)

BW698 *dhc-1(ct76ts)/unc-11(e47) dpy-5(e61)*

(Schmidt et al., 2005)

CA151 *him-8(me4)IV*

(CGC)

CA258 *zim-2(tm574)IV*

(CGC)

EU1067 *unc-119(ed3) ruIs32 [unc-119(+) pie-1^promoter^::GFP::H2B]III; ruIs57[unc-119(+) pie-1^promoter^::GFP::tubulin]*

(gift of Bruce Bowerman)

EU1561 *unc-119(ed3)III; orls17 [unc-119(+) GFP::DHC-1]; Itls37 [unc-119(+) pie-1 ^promoter^::mCHERRY::H2B]*

(Ellefson and McNally, 2011)

OD57 *unc-119(ed3)III; Itls37 [unc-119(+) pie-1 ^promoter^::mCHERRY::H2B], Itls25 [unc-119(+) pie-1 ^promoter^::GFP::tubulin]*

(McNally et al., 2006)

SMW6 strain EU1067 crossed with strain CA151 resulting in: *him-8(me4)IV; [unc-119(+) pie-1^promoter^::GFP::H2B]III; ruIs57[unc-119(+) pie-1^promoter^::GFP::tubulin]*

WH416 *unc-119(ed3) ojIs58* [*unc-119(+) pie-1^promoter^::SEP-1::GFP]III*

(Bembenek et al., 2007)

**SUPPLEMENTAL REFERENCES**

Bembenek, J.N., Richie, C.T., Squirrell, J.M., Campbell, J.M., Eliceiri, K.W., Poteryaev, D., Spang, A., Golden, A., and White, J.G. (2007). Cortical granule exocytosis in C. elegans is regulated by cell cycle components including separase. Development *134*, 3837-3848.

Ellefson, M.L., and McNally, F.J. (2011). CDK-1 inhibits meiotic spindle shortening and dynein-dependent spindle rotation in C. elegans. The Journal of cell biology *193*, 1229-1244.

Gonczy, P., Pichler, S., Kirkham, M., and Hyman, A.A. (1999). Cytoplasmic dynein is required for distinct aspects of MTOC positioning, including centrosome separation, in the one cell stage Caenorhabditis elegans embryo. J Cell Biol *147*, 135-150.

McNally, K., Audhya, A., Oegema, K., and McNally, F.J. (2006). Katanin controls mitotic and meiotic spindle length. J Cell Biol *175*, 881-891.

Schmidt, D.J., Rose, D.J., Saxton, W.M., and Strome, S. (2005). Functional analysis of cytoplasmic dynein heavy chain in Caenorhabditis elegans with fast-acting temperature-sensitive mutations. Molecular biology of the cell *16*, 1200-1212.

Vaisberg, E.A., Koonce, M.P., and McIntosh, J.R. (1993). Cytoplasmic dynein plays a role in mammalian mitotic spindle formation. J Cell Biol *123*, 849-858.

Wignall, S.M., and Villeneuve, A.M. (2009). Lateral microtubule bundles promote chromosome alignment during acentrosomal oocyte meiosis. Nature cell biology *11*, 839-844.
